# Ordinal-Level Phylogenomics of the Arthropod Class Diplopoda (Millipedes) Based on an Analysis of 221 Nuclear Protein-Coding Loci Generated Using Next-Generation Sequence Analyses

**DOI:** 10.1371/journal.pone.0079935

**Published:** 2013-11-13

**Authors:** Michael S. Brewer, Jason E. Bond

**Affiliations:** 1 Department of Environmental Science, Policy, and Management, University of California Berkeley, Berkeley, California, United States of America; 2 Department of Biology, East Carolina University, Greenville, North Carolina, United States of America; 3 Department of Biological Sciences and Auburn University Museum of Natural History, Auburn University, Auburn, Alabama, United States of America; Australian Museum, Australia

## Abstract

**Background:**

The ancient and diverse, yet understudied arthropod class Diplopoda, the millipedes, has a muddled taxonomic history. Despite having a cosmopolitan distribution and a number of unique and interesting characteristics, the group has received relatively little attention; interest in millipede systematics is low compared to taxa of comparable diversity. The existing classification of the group comprises 16 orders. Past attempts to reconstruct millipede phylogenies have suffered from a paucity of characters and included too few taxa to confidently resolve relationships and make formal nomenclatural changes. Herein, we reconstruct an ordinal-level phylogeny for the class Diplopoda using the largest character set ever assembled for the group.

**Methods:**

Transcriptomic sequences were obtained from exemplar taxa representing much of the diversity of millipede orders using second-generation (i.e., next-generation or high-throughput) sequencing. These data were subject to rigorous orthology selection and phylogenetic dataset optimization and then used to reconstruct phylogenies employing Bayesian inference and maximum likelihood optimality criteria. Ancestral reconstructions of sperm transfer appendage development (gonopods), presence of lateral defense secretion pores (ozopores), and presence of spinnerets were considered. The timings of major millipede lineage divergence points were estimated.

**Results:**

The resulting phylogeny differed from the existing classifications in a number of fundamental ways. Our phylogeny includes a grouping that has never been described (Juliformia+Merocheta+Stemmiulida), and the ancestral reconstructions suggest caution with respect to using spinnerets as a unifying characteristic for the Nematophora. Our results are shown to have significantly stronger support than previous hypotheses given our data. Our efforts represent the first step toward obtaining a well-supported and robust phylogeny of the Diplopoda that can be used to answer many questions concerning the evolution of this ancient and diverse animal group.

## Introduction

Understanding historical and contemporary patterns of biodiversity are integral to evaluating and conserving the planet’s organismal diversity. Arthropods are the largest group of animals in terms of number of nominal species, making up roughly half of all described metazoan taxa. Consequently, the higher-level classifications of many arthropod groups are understudied and have not been subjected to modern phylogenetic analysis. One such group is the subphylum Myriapoda. While the myriapod class Chilopoda (centipedes) is one of the few arthropod groups that has an ordinal level classification that presumably reflects phylogeny, is well supported, and is generally agreed upon [1]; the much larger class, the Diplopoda (Arthropoda: Myriapoda), does not [2]. 

The ancient, cosmopolitan class Diplopoda consists of primarily detritivorous species, though some have evolved additional feeding strategies (e.g., fungivory and carnivory). Despite their abundance and diversity, the class has received comparatively less attention; little is known about the group’s ecology, life histories, and evolutionary patterns and processes. In many terrestrial habitats, they are perhaps second only to terrestrial oligochaete annelids in carrying out the essential ecosystem service of breaking down dead plant matter and returning its nutrients and minerals to the soil [3]. Though generally harmless to humans, most millipedes can ward off predators by producing unpalatable defense secretions comprising a remarkable and diverse array of chemical compounds (e.g. hydrogen cyanide, benzoquinone, etc.; [4,5]). Millipedes are found on every continent (excluding Antarctica) and in virtually every biome [2,6]. The class comprises 12,000 described species with an estimated 80,000 total [7,8] (but see Brewer et al. 2012 for more conservative, empirically-derived estimates). Most taxa are described from the well-sampled temperate regions of North America and Europe, whereas less heavily sampled areas like South America currently have only six endemic families and far fewer species [Sierwald, in prep]. The group has a long evolutionary history dating back to 428 million years ago where a diplopod-like species, *Pneumodesmus newmani* Wilson and Anderson, 2004, is currently recognized as the oldest known land animal fossil [9]. A few taxa have recently been subjected to rigorous phylogenetic analyses, but these have been mostly species groups or tribes [2,10-24]. 

### Millipede Systematics

Many past attempts to classify millipedes at higher-levels have been made. Early phylogenetic studies focused on morphology and suffered from a number of shortcomings that include not employing an explicit optimality criterion [7,25-27] and a lack of sufficient taxon sampling and character breadth [2,12,13,28]. The general consensus is that all orders, except perhaps the Spirostreptida (i.e., the placement of the suborders Cambalidea and Epinannolenidea), are monophyletic, but this assumption has never been subjected to a rigorous phylogenetic evaluation [2,25]. However, the relationships between and within many of the orders are equivocal. 

The first clade to be designated within the Diplopoda was the Chilognatha Latreille, 1810. The first classification [29] focused on the 15 genera in use at the time; many of which gave rise to the currently recognized orders. After a period of considerable taxonomic proliferation in the early 20^th^ Century, Cook [26] produced a classification for 190 genera placed among 50 families. This framework was used by Hoffman [7] to produce a classification that, when translated into a phylogeny, results in a highly unresolved Helminthomorpha. Attempts to recover the phylogeny of millipedes via cladistic analyses commenced with Enghoff’s [10] morphological character-based analysis of the millipede orders. This phylogeny recovered the clade Penicillata and, as a grade, the clades Pentazonia, Helminthomorpha, Colobognatha, and Eugnatha. However, the relationships among the eugnathan orders remained unresolved. Sierwald et al. [28] used a morphological matrix derived from Enghoff [10] and included the enigmatic order Siphoniulida; their analysis recovered many of the traditional groupings. The most recent diplopod classifications are based on the works of Shelley [25] and Shear [27] both of which are derived from the nomenclatural classification scheme rather from formal analyses of data.

 The morphological characters that have been used to delimit millipede taxa vary in efficacy depending on taxonomic level. Most millipede species are described on the basis of similarities and differences in the gonopods, male modified legs used to transfer spermatophores. These character systems have been shown to be useful at the species level [30,31] but, unfortunately, are of limited use in high-level studies. Gonopods are not located on the same body region in all of the orders and do not have the same exact functional morphology; therefore they may not be homologous [2]. Beyond the gonopods, morphological character systems are group dependent when they exist; fewer than 50% of millipede higher-taxa are described using apomorphic characters [2]. 

**Figure 1:**
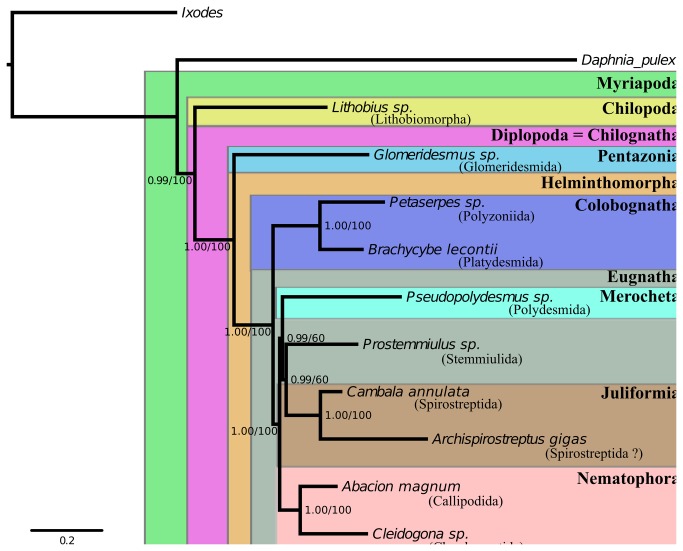
Phylogeny recovered from Bayesian inference conducted by the program Phylobayes. The traditional clades above the ordinal level are indicated by boxes. Support values are posterior probabilities/maximum likelihood boostrap values from the RAxML analysis.

More recently, phylogenetic investigations have been conducted using molecular characters to provide additional lines of evidence and testable homology hypotheses. As already mentioned above, these studies have generally lacked either sufficient loci (characters) or a sufficient number of terminals (taxa) to make definitive decisions regarding relationships and thus have resulted in often confusing, unintuitive, and unconvincing hypothesized groupings. Studies with adequate taxon sampling to address millipede relationships have only recently been attempted and focus more on the relationships within the Myriapoda [12,13]. The study published by Regier et al. [13] recovered many of the traditional orders with the Penicillata sister to the Pentazonia and Helminthomorpha. However, the tree lacks sufficient resolution at shallower levels. The most recent and only study to include both molecular data and characters based on morphology is that presented in Sierwald and Bond [2]. Combining the molecular data of Regier et al. and a novel morphological matrix comprising 41 characters, Sierwald and Bond recovered many traditional groups but found others to be polyphyletic (e.g., the Nematophora) (Figure 1). Subsequently, Brewer et al. [32], has used full mitochondrial genomes in an attempt to reconstruct the relationships between millipede ordinal taxa. The taxon sampling lacked many of the orders and most loci failed to contain adequate signal to confidently reconstruct relationships at such deep phylogenetic levels. 

 A potential source of many unlinked, protein-coding nuclear genes is information drawn from the expressed coding sequences of the millipede genome, the transcriptome. As discussed by Hedin et al. [33] next generation sequencing (NGS) technologies have provided a massive source of genomic data that can be employed for phylogenetic analyses of non-model taxa. Methods to employ these data in deep-level systematic studies are just now being developed, but a number of studies are currently available [34-36] that, taken together, provide a set of moderately well tested approaches to effectively assembling and analyzing transcriptomes for phylogeny reconstruction. 

Future detailed studies of millipede evolution and ecology require a solid classification scheme that reflects phylogenetic history. To this end, we hope to produce a well-supported ordinal-level phylogeny that is extensible to a classification reflecting the evolutionary history of these animals. The relationships both within and between many of the diplopod high-level groups are not well understood; studies focusing on ordinal level relationships using different techniques and character sources have often recovered conflicting tree topologies that support some traditional groupings while showing no evidence of others. To help elucidate the evolutionary history and relationships between the ordinal taxa of the Diplopoda, the main objective of this study is to produce a phylogeny using a massive molecular dataset assembled from coding sequences of 12 exemplar arthropod specimens (9 millipedes and 3 outgroups) representing most of the currently recognized millipede ordinal-level diversity. We employ Illumina RNA-Seq data to generate the first ever phylogenomic data set produced for a myriapod taxon.

## Materials and Methods

### Taxon Sampling, RNA Isolation, and High-Throughput Sequencing

 No special permits were required for the described field studies, the locations were not privately owned or protected, and the study organisms are not endangered or protected. Taxa were targeted to represent the diversity of ordinal level taxa of the Diplopoda and a centipede outgroup (Table 1). Living animals were field collected and preserved in RNAlater (Qiagen, Inc, Valencia, CA) either immediately or upon return to the lab. The anterior head region plus several subsequent body rings were extracted from larger animals, frozen in liquid nitrogen, and crushed with a mortar and pestle. Total RNA was extracted using the RNEasy extraction kit and Shredder columns (Qiagen, Inc, Valencia, CA). The entire bodies of smaller animals were used. Total RNA was quantified and shipped to Hudson Alpha (Huntsville, Alabama) on dry ice for cDNA library preparation and subsequent sequencing. The barcoded libraries were pooled four to a flowcell lane and sequenced using the Illumina RNA-seq method with HiSEQ Paired-end 50 bp chemistry. Voucher specimens are available from the Auburn University Museum of Natural History collection, and the sequence data are available from the NCBI short-read archive (SRA) (accession s: SRX326775 – SRX326777, SRX326779 – SRX326784).

**Table 1:** Illumina RNA-seq, quality control, and HaMSTR results.

**Exemplar**	**# of raw reads**	**# of processed reads**	**# of contigs**	**# of HaMSTR orthologs**
*Lithobius*	63,859,222	46,366,497	33,692	877
*Glomeridesmus*	30,728,054	23,473,057	20,181	885
*Petaserpes*	43,543,711	32,494,393	12,154	715
*Brachycybe*	34,242,917	26,009,589	18,570	924
*Pseudopolydesmus*	40,382,211	30,638,665	18,876	909
*Prostemmiulus*	41,343,098	29,259,335	10,524	594
*Cambala*	38,623,633	27,334,077	14,071	755
*Abacion*	39,893,805	30,238,012	15,861	755
*Cleidogona*	29,767,350	22,288,076	16,572	877
Archispirostreptus*	N/A	N/A	4,008*	169
Ixodes*	N/A	N/A	38,392*	815

The numbers of reads pre- and post-processing for each taxon with the FASTX Toolkit are shown here. Additionally, the numbers of HaMSTR orthologs for each taxon is listed. * indicates taxa that were not sequenced as part of this study.

### Quality Control, Sequence Assembly, and Coverage Estimation

 The resulting reads for each taxon were contained in two FASTQ files representing each of the paired-end reads; primer and barcode sequences were removed immediately after sequencing. Individual read files were subjected to quality trimming using the FASTX Toolkit [37]. All sites occurring after a position with a quality score of 20 or less were removed, and all sequences less than 30 bases in length were deleted. The first nine bases of each sequence were removed to eliminate primer artifacts. The reads were resynchronized using the script sync_paired_end_reads.py (https://github.com/martijnvermaat/bio-playground/tree/master/sync-paired-end-reads). The cleaned files were examined for anomalies and contamination using FASTQC [38]. 

 Contigs representing unique mRNA transcripts were assembled using the Trinity pipeline [39]. The following parameters were used: min_kmer_cov 2, run_butterfly, CPU 6, bflyHeapSpace 10G. 

 Transcriptome coverage estimation was assessed by comparing each assembly to a standard set of 248 core eukaryotic genes (CEGs) using the program Cegma [40]. These CEGs were derived from the eukaryotic orthologous groups (KOGs), a subset of the Cluster of Orthologous Groups (COGs) of Proteins database [41].

### Orthology Assessment and Dataset Construction

 Trinity output representing assembled transcripts and EST contigs from GenBank (*Ixodes scapularis* Say, 1821 and *Archispirostreptus gigas* (Peters, 1855)) were analyzed using the HaMStR [42] approach to identify orthologs. The HaMStR method uses “core orthologs” from a defined set of proteomes to train Hidden Markov Models (HMMs) to detect orthologous sequences in Expressed Sequence Tag (EST) libraries or transcriptomes. The arthropod core orthology set was employed, and *Daphnia pulex* (Linnaeus, 1758) (Arthropoda: Pancrustacea: Branchiopoda) was used as the first reference taxon because it is presumably the closest relative to the myriapods and arachnids in the provided sequences. The default e-value was changed to “1e-20” to be more conservative (although the total number of orthologs recovered varied little when using the default of “1”). The representative option, which concatenates contigs representing non-overlapping sections of a single ortholog, was used to increase data recovery. 

 Ortholog-specific FASTA files were assembled representing sequences from all taxa for which the locus of interest was recovered by the HaMSTR method. All reference taxa were removed from these individual ortholog files except *Daphnia pulex* to increase the ratio of ingroup to outgroup taxa. Custom scripts were used to trim any ambiguous sequence from the first twenty bases of each sequence of all ortholog files. The resulting FASTA files were individually aligned using MAFFT [43,44]. The program SCaFoS [45] was used to select alignments for phylogenetic analysis. The following SCaFoS parameters were used gamma=yes, puz=yes, t=5, o=gscl, s=50, g=50, m=25, format=fpmnba, color=yes, cmp=yes. Gblocks [46,47] was used to trim misaligned areas or areas with many indels with the following parameters: -b1=”half of sequences in the alignment +1”, -b2=”half of sequences in the alignment +1”, -b3=8, -b4=10, -b5=h. Any remaining columns containing all gaps or only one non-gap character were deleted from all alignments. In an attempt to remove any paralogous sequences that were falsely included, any sequence in which 75% or more amino acid residues differ from the consensus were deleted using output from the EMBOSS package infoalign [48]. Alignment masking was performed on each remaining ortholog alignment using Aliscore version 1.0 [49] and ALICUT version 2.0 [50]. Any remaining alignments less than 100 AA in length were deleted, individual sequences shorter than 100 AA were deleted, and alignments with less than 11 of the 12 included taxa were deleted. The cutoff for taxon inclusion (11 of 13) was chosen to be the most conservative while recognizing the limitations of the EST dataset (i.e., *Archispirostreptus*). The final processed alignments were concatenated using FASconCAT [51] for subsequent supermatrix analyses. 

### Phylogenetic Inference

 Phylogenetic trees were inferred using maximum likelihood (ML) and Bayesian inference (BI) optimality criteria. The ML trees were obtained via analyses of the individual alignments and the supermatrix with partitions corresponding to each ortholog in RAxML version 7.2.8 [52]. Analyses consisted of 1,000 random addition sequence replicates (RAS) with support values obtained from 1,000 bootstrap replicates. The PROTGAMMAWAG model of AA substitution was used for all partitions. BI trees based on the supermatrix were obtained using Phylobayes version 3.3b [53]. Five independent chains were run for 10,000 cycles sampling every cycle analyzing the supermatrix using default parameters. Run convergence was estimated and consensus trees were obtained using the bpcomp command. The first 20% of cycles were discarded as burn-in. To assess the sensitivity of our resulting phylogeny to long-branch attraction artifacts and outgroup selection, we removed the outgroup taxa and reran the phylogenetic analyses as above.

 To assess whether our results are significantly better than previous estimates of millipede ordinal phylogeny given our novel data, we conducted likelihood-based topology tests. Previous tree topology hypotheses that were well-resolved in regards to the taxa included herein were trimmed to contain only those taxa present in our study [2,10,54,55]. Likelihood values for all topologies were calculated using FastTree 2.1 [56,57] and were compared using CONSEL [58]. CONSEL employs eight methods for comparing tree topology hypotheses given a dataset: approximately unbiased test (AU), two bootstrapping methods (NP & BP), Bayesian posterior probabilities (PP), Kishino-Hasegawa (KH), Shimodaira-Hasegawa (SH), weighted Kishino-Hasegawa (WSH), and weighted Shimodaira-Hasegawa (WSH).

### Ancestral State Reconstruction

 The BI phylogeny and Mesquite [59] were used to reconstruct the ancestral states of three characters of importance in millipede evolution: gonopods, ozopores, and spinnerets. The characters were coded as discrete and unordered as follows for the orders the exemplar represents: gonopods (0=none, 1=ninth and tenth leg pairs, and 2=eighth and ninth leg pairs), ozopores (0=none, 1=present), and spinnerets (0=none, 1=present). A parsimony model was used to reconstruct the ancestral states at all nodes on the tree.

### Molecular Divergence Dating

 Estimates of divergence dates and 95% confidence intervals were obtained using Phylobayes 3.3 and the majority rule consensus tree from the BI analyses described above. Two fossil constraints were used, *Pneumodesmus newmani* Wilson and Anderson, 2004 (origin of the Helminthomorpha - 428 MYA [9]) and *Sigmastria dilata* (origin of the Juliformia - 410 MYA [60]). A maximum age for the origin of the Diplopoda was set to coincide with the emergence of land plants (~480 MYA), and the age of the root node was set at 560 MYA, following Rehm et al. [61] with an exponential distribution (i.e., a standard deviation of 560 MY). The analysis was run for 10,000 cycles under the “UGAM” method [62] and for 10,000 cycles under a lognormal relaxed clock model. The first 2,000 generations of each run were discarded as burn-in.

## Results

### Transcriptomic sequences and dataset assembly

 The numbers of reads before quality control filtering, the numbers of reads after filtering, the numbers of contigs, and the numbers HaMSTR orthologs for each taxon are summarized in Table 2. Following quality screening, an average of 74.18% (70.77% - 76.39%) of reads were retained. The average number of Trinity assembled contigs for our novel transcriptomes was 17,833.44 (10,524 - 33,692). This average is highly skewed due to the relatively large number of *Lithobius* sequences. *Lithobius* sequences were generated independently in a separate Illumina RNA-seq run. Estimates of transcriptome completeness are summarized in Table 3. Complete protein coverage estimates range from 33.47% (*Prostemmiulus* sp.) to 81.85% (*Brachycybe lecontii*) with a mean of 57.48%, and partial coverage estimates range from 56.85% (*Prostemmiulus* sp.) to 93.95% (*Brachycybe lecontii*) with a mean of 73.93%. The mean number of HaMSTR orthologs recovered for our newly sequenced taxa was 810.11 (594 - 924). 

**Table 2:** The numbers of loci, characters, gaps, and missing data (i.e., ? or X) are listed for pre- and post-dataset optimization.

	**Pre-optimization**	**Post-optimization**
# of loci (HaMSTR orthologs)	1005	221
# of characters (aligned AAs columns)	532,002	61,641
# of gaps (% of data)	1,788,558 (28.02%)	53,286 (7.20%)
Missing data (% of data)	1,833,905 (28.73%)	75,146 (10.16%)

Optimization included getting rid of short sequences (<200 AAs), removing individual gene alignments with less than 11 taxa, locus selection using SCaFoS, running GBlocks on each alignment to remove overly “gappy” regions, and alignment masking with Aliscore and ALICUT.

**Table 3:** Cegma transcriptome completeness estimation results.

	Complete Coverage		Partial Coverage	
Taxon	**No. of Proteins**	**Percentage of Completeness**	**No. of Proteins**	**Percentage of Completeness**
*Abacion magnum*	132	53.23	180	72.58
*Brachycybe lecontii*	203	81.85	233	93.95
*Cambala annulata*	139	56.05	173	69.76
Cleidogona sp.	136	54.84	171	68.95
Glomeridesmida sp.	159	64.11	200	80.65
Lithobius sp.	154	62.1	188	75.81
Petaserpes sp.	120	48.39	173	69.76
Prostemmiulus sp.	83	33.47	141	56.85
Pseudopolydesmus sp.	157	63.31	191	77.02
Mean:	**142.56**	**57.48**	**183.33**	**73.93**

For each taxon, the number of 248 possible CEG proteins recovered in the transcriptome assembly and the resulting percentage is shown for both complete and partial coverage.

 The numbers of loci, aligned amino acid residues, gaps, and missing data for the HaMSTR orthologs are summarized in Table 4. HaMSTR identified a total of 1,005 unique orthologs in all taxa examined (novel sequences plus those obtained from GenBank). Alignments of these loci contained 532,002 independent amino acid sites. Among these 1,788,558 gaps were present and 1,833,905 positions were missing data (the locus was not recovered for one or more taxa). Following phylogenetic dataset optimization (i.e., gap removal, locus selection, per locus taxon selection, alignment masking, and alignment size filtering), 221 loci remained and were used in subsequent analyses. In total, 61,641 aligned sites were used to reconstruct evolutionary relationships. The number of gaps in the final concatenated dataset was 53,286, and 75,146 positions were missing data. The levels of inclusion for each taxon are summarized in Table 5. On average, each taxon was present in 88.15% of loci in the final dataset.

**Table 4:** Levels of taxon inclusion pre- and post-dataset optimization.

Taxon	**Pre-optimization**	**Post-optimization**
*Lithobius*	878 (87.36%)	214 (96.83%)
*Glomeridesmus*	886 (88.16%)	218 (98.64%)
*Petaserpes*	716 (71.24%)	204 (92.31%)
*Brachycybe*	925 (92.04%)	220 (99.56%)
*Pseudopolydesmus*	910 (90.55%)	216 (97.74%)
*Prostemmiulus*	595 (59.20%)	186 (84.16%)
*Cambala*	756 (75.22%)	216 (97.74%)
*Abacion*	756 (75.22%)	217 (98.19%)
*Cleidogona*	759 (75.52%)	211 (95.48%)
Archispirostreptus*	170 (16.92%)	33 (14.93%)
Ixodes*	816 (81.19%)	208 (94.12%)

Optimization included getting rid of short sequences (<200 AAs), removing individual gene alignments with less than 11 taxa, locus selection using SCaFoS, running GBlocks on each alignment to remove overly “gappy” regions, and alignment masking with Aliscore and ALICUT. * indicates taxa that were not sequenced as part of this study.

**Table 5:** Currently millipede classification scheme, adopted from Shear [27].

**Class**	**Subclass**	**Infraclass**	**Subterclass**	**Superorder**	**Order**
Diplopoda (millipedes)	Penicillata				Polyxenida (bristly millipedes)
	Chilognatha	Pentazonia (pill millipedes)		Limacomorpha	Glomeridesmida
				Oniscomorpha	Glomerida
					Sphaerotheriida
		Helminthomorpha (worm-like millipedes)	Colobognatha		Platydesmida
					Polyzoniida
					Siphonocryptida
					Siphonophorida
			Eugnatha	Merocheta	Polydesmida
				Nematophora	Stemmiulida
					Callipodida
					Chordeumatida
					Siphoniulida
				Juliformia	Julida
					Spirobolida
					Spirostreptida

### Phylogenetic inference

 The resulting phylogenies had overall strong support values. The trees obtained from the ML and BI searches were similar in all but the placement of one terminal, *Pseudopolydemsus* sp. The ML analysis phylogeny places the Polydesmida (*Pseudopolydesmus* sp.) at the base of the Helminthomorpha while the BI analysis recovered the terminal at the base of the Juliformia+Stemmiulida. The support values in the ML tree are low in the nodes involving these taxa. The BI posterior probabilities are all very high (0.99 or 1.00 in all cases), and the ML bootstrap analysis supports the BI tree better than the ML best tree. Both analyses recovered a monophyletic Myriapoda, Diplopoda, Helminthomorpha, Colobognatha, Eugnatha, and Juliformia. The Nematophora (traditionally Stemmiulida, Callipodida, and Chordeumatida) was not recovered. The Stemmiulida (*Prostemmiulus* sp.) was recovered as sister to the Juliformia in both analyses.

**Figure 2:**
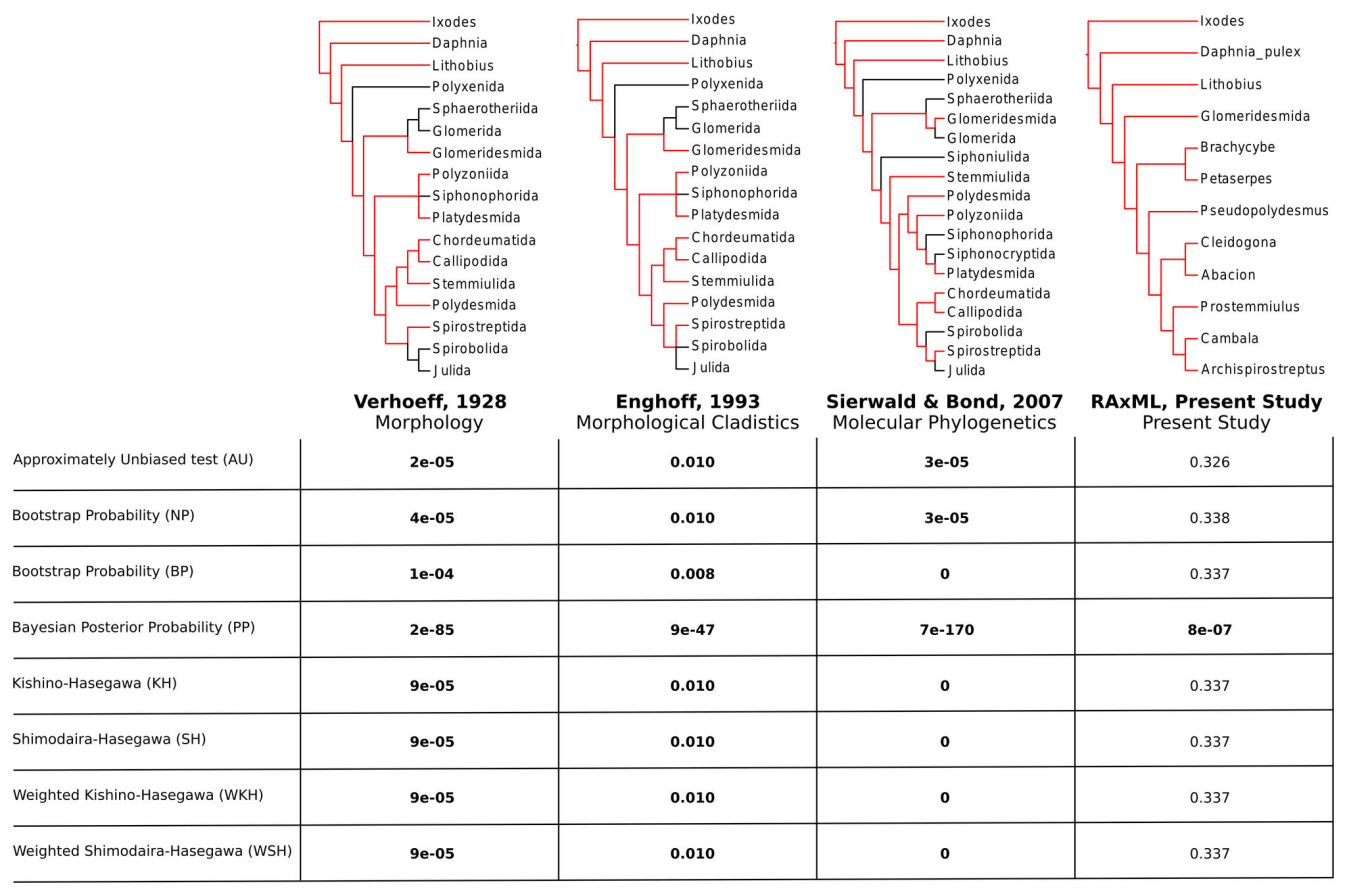
Topology comparisons between our Phylobayes results and past studies. The phylogeny obtained from phylobayes was compared to past hypotheses that were well-resolved regarding our included taxa. Three topologies met our criteria of taxon inclusion and resolution: Verhoeff [54], Enghoff [10,55], and Sierwald and Bond [2]. The full trees from each study are shown, the data source used to build the tree is indicated, and branches leading to taxa included in our analysis are shown in red. P-values obtained from each test are provided. Significant results (p < 0.05) are bolded and indicate the likelihood value of our Phylobayes results were significantly better than the alternative. Our RAxML results were also compared to the Phylobayes tree.

 The phylogeny of the Diplopoda (ingroup) did not change and support values were unaffected when outgroups were removed. Comparisons to previous hypotheses all supported our preferred phylogeny regardless of the comparison method (Figure 2). Only a single method (Bayesian posterior probabilities) could distinguish between our Phylobayes and RAxML topologies and preferred the Phylobayes results.

### Ancestral State Reconstructions

**Figure 3:**
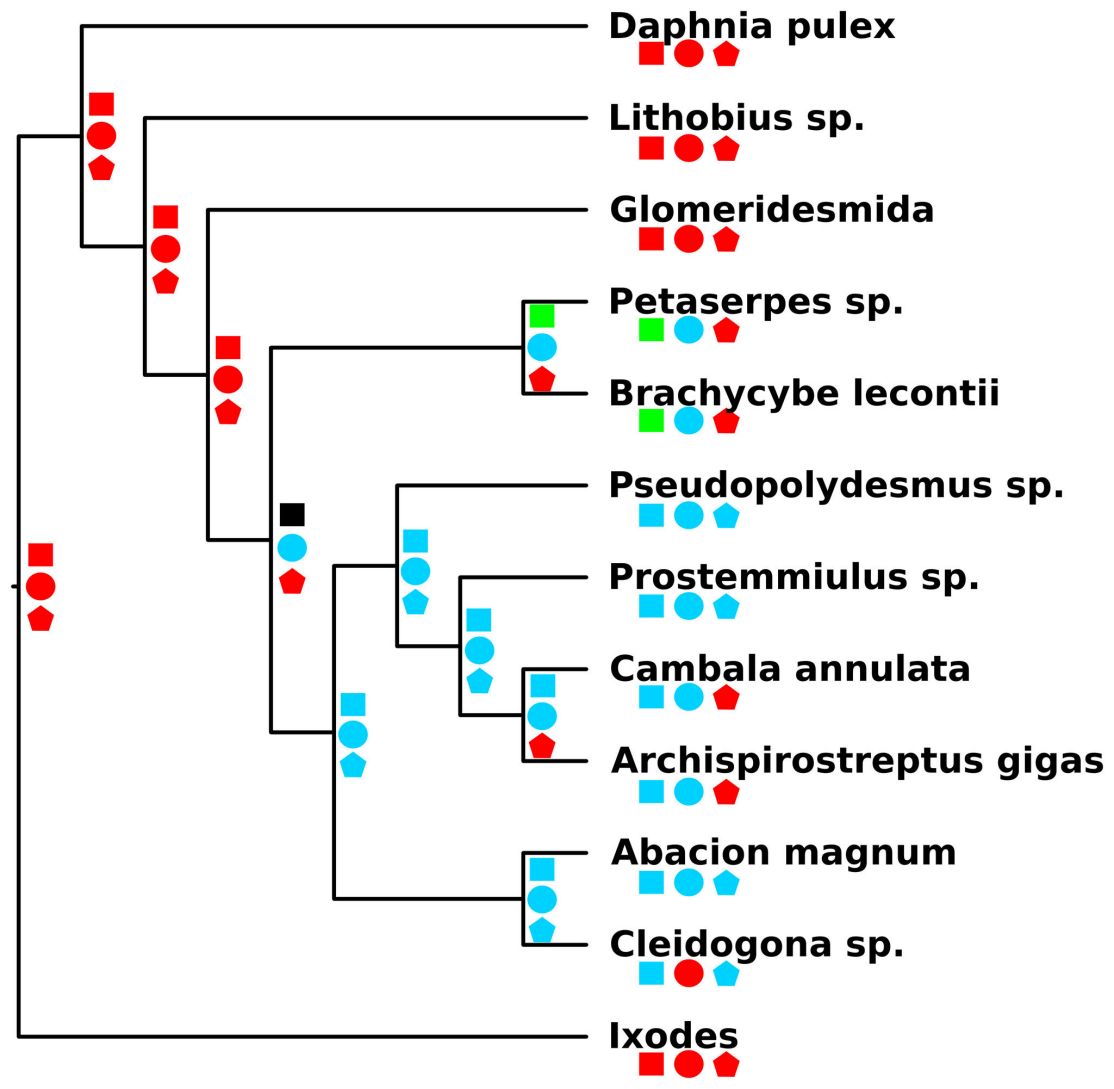
Ancestral character state reconstructions of features relating to millipede gonopods, ozopores, and spinnerets. The phylogeny obtained from phylobayes imported into Mesquite and states were reconstructed under a parsimony model. Squares correspond to gonopods (red = none, green = ninth and tenth legs pairs, blue = eighth and ninth leg pairs, black = ambiguous). Circles correspond to the presence of millipede ozopores (red = none and blue = present). Pentagons correspond to millipede spinnerets (red = none and blue = present).

 The results of the Mesquite ancestral reconstructions are summarized in Figure 3. Gonopods originating from the ninth and tenth leg pairs were inferred to have been present in the ancestor of the Colobognatha. Gonopods stemming from the eighth and ninth leg pairs were inferred to have been present in the ancestor of the Eugnatha and all daughter nodes. Ozopores, and subsequently repugnatory secretions, were reconstructed in the ancestor of the Helminthomorpha and all subsequent nodes. Finally, spinnerets were inferred to have been present in the ancestor of the Eugnatha and all daughter nodes excluding the Juliformia.

### Molecular divergence dating

**Figure 4:**
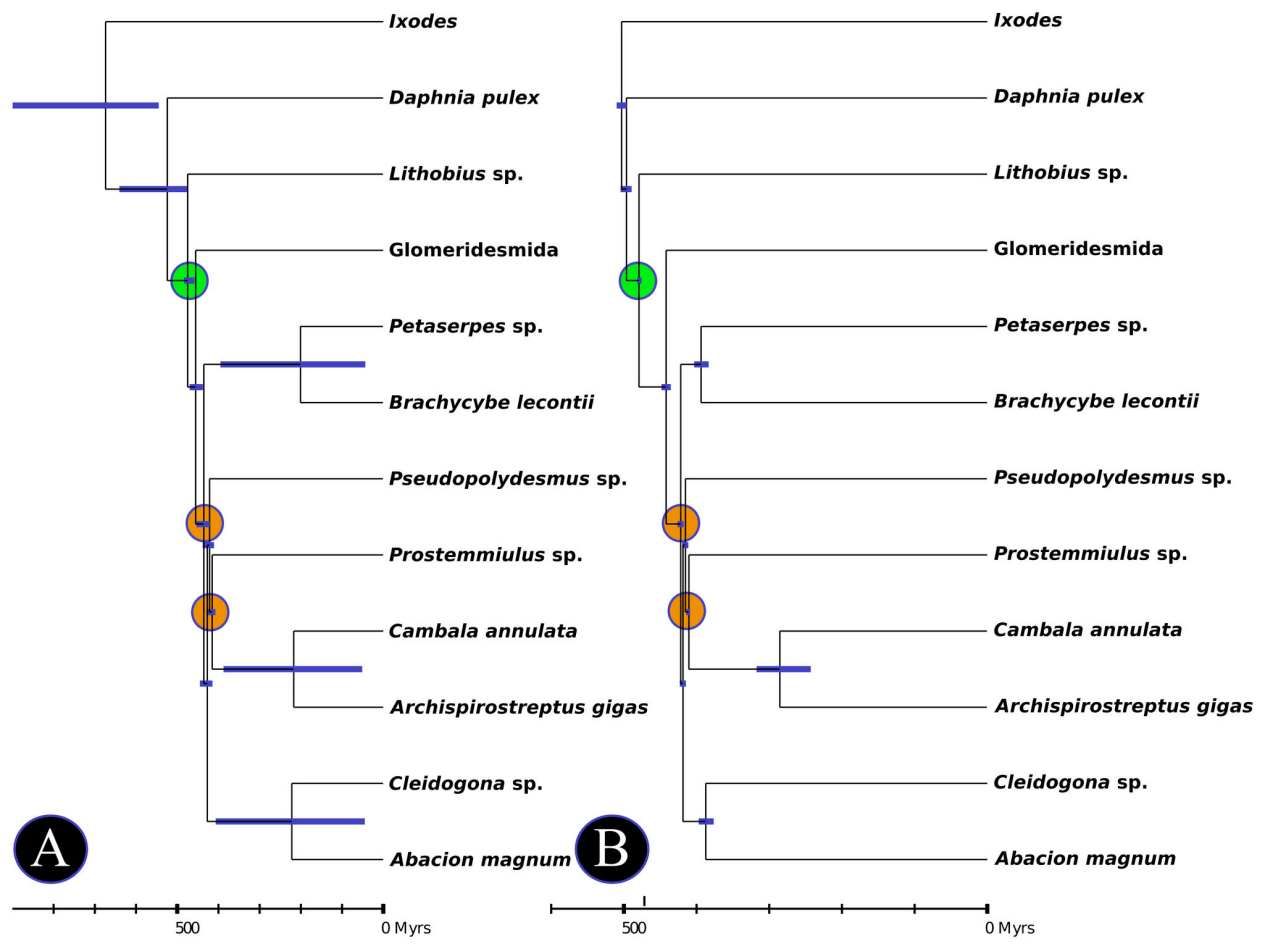
Chronograms representing the estimated divergence times for the lineages included in this study. All analyses were conducted in Phylobayes using the Bayesian inference topology. A single fossil constraint was used, *Pneumodesmus newmani* - ~428 MYA, and the root prior was set at 510 MYA (as estimated by Rehm et al. [61]) with an exponential distribution. The orange circles indicate the fossil constraints, and the green circle indicates maximum age constraint placed on the Diplopoda (the emergence of land plants). A) divergence times estimated using the “UGAM” model; B) divergence times estimated using the “log normal” model.

 The divergence timing estimates are summarized in Figure 4. The two models used to estimate the timing of divergence between clades recovered in the BI tree recovered similar results. The UGAM results generally had wider confidence intervals and older point estimates. The nodes near to the fossil calibration point were more similar between the two analyses and had tighter confidence intervals under both models. 

 The uncorrelated gamma method [62] (Figure 4A) recovered the origin of the Mandibulata (i.e., the split from the Chelicerata) as 674.182 MYA. The origin of the Myriapoda was estimated to have occurred 523.724 MYA. Within the Diplopoda (estimated to have split from the Chilopoda 474.489 MY in age), the following dates were recovered for groups: Pentazonia/Helminthomorpha = 455.003 (fossil constraint), Colobognatha/Eugnatha = 435.397 MYA, Polyzoniida/Platydesmida = 200.751 MYA, Coelocheta/Juliformia+Polydesmida+Stemmiulida = 426.971 MYA, Chordeumatida/Callipodida = 221.541 MYA, Polydesmida/Juliformia+Stemmiulida = 421.263, Stemmiulida/Juliformia = 414.728 MYA (fossil constraint), and Cambalidea/Spirostreptidea = 217.137 MYA.

 The lognormal model recovered younger ages and tighter confidence intervals at deep nodes than the UGAM analysis. The Mandibulata was inferred to be 503.322 MY in age. The origin of the Myriapoda was estimated to have occurred 496.472 MYA. Within the Dipolopoda (estimated to have split from the Chilopoda 479.166 MYA), the following dates were recovered for groups: Pentazonia/Helminthomorpha = 441.905, Colobognatha/Eugnatha = 421.879 MYA (fossil constraint), Polyzoniida/Platydesmida = 393.646 MYA, Coelocheta/Juliformia+Polydesmida+Stemmiulida = 418.679 MYA, Chordeumatida/Callipodida = 387.323 MYA, Polydesmida/Juliformia+Stemmiulida = 415.351, Stemmiulida/Juliformia = 410.718 MYA (fossil constraint), and Cambalidea/Spirostreptidea = 285.538 MYA.

## Discussion

### Transcriptomic sequences and dataset assembly

 High throughput sequencing technologies provide millions of reads that confer high confidence in base calls and, when assembled, data from thousands of loci. The use of transcriptomic data allow us to construct datasets comprising many unlinked regions of millipede genomes comprising only protein-coding sequence, which are more likely to be appropriate for deep phylogenetic studies. By doing so, we can confidently avoid the impact of past hybridization events and deep coalescent problems that can have profound effects on phylogenetic studies that have previously relied on mitochondrial gene regions or just a few nuclear loci [63]. Additionally, such a tremendous wealth of data permits us to be selective with respect to loci included in phylogenetic analyses while still assembling relatively large datasets. The HaMSTR approach of choosing orthologs from EST data, or RNA-seq data in our case, performed adequately. By targeting core orthologs, the difficulties associated with *de novo* orthology assessment and varying completeness of transcriptome recovery are largely alleviated.

 The HaMSTR approach coupled with various levels of dataset optimization has been successfully employed in other recent studies of scorpions [34] protostomia [46,47] arthropods [64], and molluscs [65]. These studies all employed some varied combination of both traditional and second generation sequencing techniques and a range of dataset optimization approaches, but used similar approaches to filtering data for orthologous sequences. While Illumina reads lengths are currently considerable shorter than those produced by the 454 pyrosequencing technology, these methods are more cost effective, yield more data, and are well-suited for transcriptome sequencing due to the lack of homopolymers in most coding DNA. 

 By using dataset optimization techniques on individual gene alignments, we reduced the total millipede dataset to 11.59% of its original concatenated size. These methods reduced the occurrence of gaps in the alignments by 20.82% and missing data by 18.57%. Our data were optimized, with regards to signal and information content, using only the taxa included in this study. Although a lot of data were discarded, these rejected alignment regions were found to contain too many gaps or lacked appropriate phylogenetic signal. The optimization methods allowed us to build datasets that comprise confidently aligned orthologs that are relatively free of phylogenetic “noise”. In the end, this highly filtered data set comprised 221 nuclear protein coding genes composed of 61,641 aligned amino acid residues (Table 2).

### Millipede phylogenomics

 The phylogeny obtained from the BI analysis (Figure 1) has high support values at many nodes and recovered many groupings supported in past analyses [2,13]. The Myriapoda was recovered as monophyletic with a 0.99 posterior probability. The Diplopoda (or, more accurately, the Chilognatha) was recovered with a posterior probability of 1.00. Because no members of the order Polyxenida were included in the analyses, the millipede clade strictly depicts the Chilognatha (Pentazonia + Helminthomorpha). Within the Helminthomorpha (worm-like millipedes), the Colobognatha and Eugnatha were each recovered with posterior probabilities of 1.00. This is significant because past analyses of morphology and molecules [2,13] do not recover a monophyletic Eugnatha. The Nematophora (Stemmiulida+Callipodida+Chrodeumatida) was not recovered *sensu stricto* as the Stemmiulida allied with the Juliformia and Polydesmida (PP = 0.99). The Stemmiulida has been separated from the other nematophorans in past analyses [2,13] and was not included in the group by Hoffman [7]. To our knowledge, a grouping of Juliformia+Polydesmida+Stemmiulida has never before been proposed. 

 The ML phylogeny was similar to the BI tree except with regards to the placement of the Polydesmida. The polydesmidan exemplar, *Pseudopolydesmus* sp., was recovered as basal to the remaining eugnathan millipedes in the ML analysis. The support for the remaining eugnathan orders as a monophyletic group excluding the Polydesmida had low support (BS = 20). When the ML bootstrap replicates were used to assess support for the BI tree (i.e., support for the BI tree was calculated in RAxML using the previously generated bootstrap results), the Phylobayes topology had greater support at all nodes than the RAxML tree. In general, the BI tree had better support than the ML tree and is our preferred topology. 

 The dataset presented here appears to be robust regarding outgroup selection. The topology was unaffected when the outgroup taxa were removed indicating strong signal in the data. Additionally, the data support our results over previous hypotheses regardless of the comparison method used (Figure 2). Unfortunately, only a single method preferred our Phylobayes results over the RAxML trees, and all other methods could not distinguish between them.

### A consideration of millipede classification

 The current millipede classification [27], summarized in Table 5, comprises 16 extant orders placed in a number of superordinal and higher taxa. The class Diplopoda, the millipedes, is universally considered a natural group and is defined by four synapomorphies: 1) body segments are fused to form diplosegments in the trunk region, resulting in two leg pairs per body segment for most of the length of the body; 2) the first segment behind the head, the collum, region is legless – the class Pauropoda, the presumed sister group to the millipedes, has a collum segment, but this segment has leg rudiments; 3) the antennae of millipedes bear four sensory cones; and 4) sperm are aflagellate. The BI and ML phylogenies clearly support a monophyletic Diplopoda (although our analyses do not include the millipede order Polyxenida or the sister class Pauropoda), as do previous cladistic and molecular phylogenetic analyses of the group [2,10,12,13,28].

 The most “primitive” millipede order, the Polyxenida (bristly millipedes), is the sole member of the subclass Penicillata. Polyxenidans lack modified mating appendages of any kind and, instead, deposit spermatophors on a substrate for the females to find and pick up. Additionally, bristly millipedes do not produce defense secretions but instead have long modified setae that break off and tangle the mandibles of predatory insects [66]. The group is defined by having tufts of setae and a transverse suture between the ocelli and antennae; their bodies are generally soft and uncalcified. The Tömösváry organ is small in most species. Although no bristly millipedes were included in our analyses, the order Polyxenida was the sister group to the remaining millipedes in Sierwald and Bond [2], Sierwald et al., and Enghoff [10] and thus is likely to remain non-contentious. However, Regier and Shultz [12] and Regier et al. [13] recovered a Polyxenida+Pentazonia clade in some of their analyses.

 The remaining millipede orders are placed in the subclass Chilognatha comprising the Pentazonia (pill millipedes) and Helminthomorpha (worm-like millipedes) clades. The subclass is composed of 15 orders and is defined by three synapomorphies: 1) calcified cuticle; 2) the absence of trichobothria; and 3) sternites and first legs fused in females. All millipede transcriptomes used to reconstruct the evolutionary relationships reported herein represent Chilognatha taxa; the group is recovered as monphyletic in all of our analyses (Figure 1). Of the published millipede phylogenetic studies, only Regier and Shultz [12] and Regier et al. [13] did not recover a monophyletic Chilognatha. As mentioned above, the Polyxenida rendered the Pentazonia paraphyletic, thus calling into question the monophyly of the Chilognatha. Alternatively, the ML analysis of Regier et al. [13] finds Chilognatha to be monophyletic.

 The infraclass Pentazonia comprises three orders arranged into two superorders: the Linacomorpha (order Glomeridesmida) and the Oniscomorpha (orders Sphaerotheriidia and Glomerida). Pill millipedes generally have modified posterior appendages in males for grasping females during mating and lack defense secretions (although the Glomerida may have evolved chemical defenses independent of the remaining diplopod taxa). The following five characters define the Pentazonia: 1) divided sternites; 2) a labrum that has a single median tooth; 3) lamellae lingules of the gnathochilarium fused; 4) mandible with molar plate process; and 5) the last tergite is enlarged and covers the anal segment. Because we included only a single pill millipede (*Glomeridesmus* sp.) in our analysis, we cannot comment on the monophyly of the group as a whole. While most analyses support the monophyly of the Pentazonia, Regier and Shultz [12] recovered a tree that calls the monophyly of the pill millipedes into question. The Limacomorpha (= order Glomeridesmida) is distinguished by the following characteristics: 1) females with pleated ovipositor at coxa of leg pair 2 and 2) last pair of legs held straight out posteriorly. The Oniscomorpha comprises the two orders Glomerida and Sphaerotheriida. The group is defined by having only up to 13 body rings. Of the published studies that include adequate sampling of pill millipedes [2,12,13,20,28], only Regier and Shultz [12] disputed the monophyly of the Oniscomorpha.

 The infraclass Helminthomorpha contains the remaining 12 orders in two subterclasses, the Colobognatha and Eugnatha. Helminthomorpha are characterized by: 1) the presence of paired lateral ozopores (pores for release of repugnatory secretions) on each trunk diplosegment starting at body ring five, 2) lacking spiracles on body rings two and three, and 3) the tracheae are not branched (as in the Polyxenida and Pentazonia). Species in the Helminthomorpha also possess modified anterior legs in males, the gonopods, to transfer a spermatophore to the female during mating. Our results (Figure 1) and all other published phylogenetic studies support Helminthomorpha monophyly [2,10,12,13,28]. When the presence of ozopores was reconstructed on the BI tree (Figure 3), the use of repugnatory secretions appears to have been present in the ancestor of the Helminthomorpha and was subsequently lost in the Chordeumatida and Siphoniulida, although the latter is of contentious phylogenetic placement. 

 The subterclass Colobognatha comprises four orders: Platydesmida, Polyzoniida, Siphonocryptida, and Siphonophorida. Colobognathans are recognized as having the following characteristics: 1) simple leg-like gonopods (two pairs) on body rings seven and eight that modify step-wise from walking legs as the animals mature, 2) palps associated with the gnathochilarium absent, and 3) tubular repugnatory glands. Colobognathan gonopods have historically been thought to offer little taxonomic information, even at the species level [2,67]. Our analyses support a Colobognathan clade sister to the remaining Helminthomorpha (=Eugnatha) (Figure 1) as does Sierwald et al. [28] and Enghoff [10]. However, few other phylogenetic analyses have recovered this grouping and usually ally the Polydesmida with the Colobognatha, thus rendering the Eugnatha paraphyletic [2,12,13].

 The subterclass Eugnatha comprises eight orders and contains the vast majority of millipede species diversity (~11,000 of ~12,000 nominal species). Eugnathans often have highly modified gonopods that are diagnostic at lower taxonomic levels. The group is currently split into three superorders: Merocheta, Nematophora, and Juliformia. The Eugnatha is defined by the following synapomorphies: leg pair eight modified into gonopods, gonopods develop from small bud-like structures, tergites and pleurites fused, and globular defense glands. The sister relationship between the Colobognatha and a monophyletic Eugnatha is supported by Sierwald et al. [28], Enghoff [10], and in both our BI and ML trees (Figure 1). Characteristics relating to the development of gonopods between the two groups appear to reinforce their exclusivity. The gonopods of colobognathans and eugnathans are, however, of questionable homology [2]. Our ancestral state reconstruction of gonopod developmental origins recovered an ambiguous condition for the ancestor to the Helminthomorpha (Figure 3), thus illustrating the difficulty in assigning homology to these structures.

 The Merocheta contains a single, yet very diverse (>5,000 nominal species), order, Polydesmida. Polydesmidans are defined as having between 19 and 21 body rings, a projection at the seventh antennomere, fused sternites lacking a suture, no ocelli, first gonopods with cannula, no ozopores on body ring six, two compartments in the repugnatory glands, cyanide in the defense secretions. Polydesmidan species may have functional spinnerets [68]. These species can be quite colorful and often have wing-like paranota extending laterally from the tergites. The monophyly of the Polydesmida is unequivocal.

 Three orders, each possessing posterior structures often considered anecdotally to be “silk spinnerets”, make up the Nematophora: Stemmiulida, Chordeumatida, and Callipodida. Nematophorans are defined as having spinnerets on the telson, branched tracheae associated with the spinnerets, and a molar cusp at the mandible. The Chordeumatida are the only traditional eugnathan order to lack defense secretions (the Siphoniulida have only recently been placed in the Eugnatha). The Stemmiulida is currently of controversial placement [2] and has been said to not closely ally with any extant Eugnathan group [69]. As mentioned above, the Stemmiulida were placed in a clade with the Polydesmida and Juliformia in our BI and ML phylogenies. The trees presented herein recover a monophyletic Coelocheta (Callipodida+Chrodeumatida). This grouping was described by Hoffman [7] and is currently defined as follows: unique Tömösváry organ morphology and mentum of gnathochilarium transversely divided. The monophyly of the Coelocheta, a clade not recognized by Shelley [25] or Shear [27], has been supported in other studies [2,13,28]. The use of spinnerets to define the Nematophora is difficult given that many other groups produce “silk”, and some even have spinnerets [68]. Therefore, the homology of these structures is difficult to ascertain, and their presence/absence could have multiple meanings. Either there have been several independent originations of silk spinning/spinnerets (i.e., convergent evolution or homoplasy), or spinnerets originated once and were subsequently lost in some lineages. We reconstructed the ancestral conditions for putative millipede spinnerets on the BI phylogeny (Figure 3) and found that the ancestor of all eugnthans likely had spinnerets. These structures were subsequently lost in the Juliformia based on this character transformation and may represent a putative synapomorphy for the group. 

 The order Siphoniulida has proven difficult to place. Currently the group is considered a member of the Nematophora [27] based on spinneret structures. The order is extremely rare; males were described for the first time in 2003 [28]. Siphoniulidans have fused sternites, no ozopores, no ocelli, and unique characteristics of the gonopods stemming from leg pair eight. Leg pair nine is unmodified in males and the Tömösváry organ is absent. The animals have structures identical in form to the spinnerets of stemmiulidans. Siphoniulidans are not represented in this study, and only two past phylogenetic studies have attempted to place them on the millipede tree of life; Sierwald et al. [28] was the first. Sierwald and Bond [2] included the Siphoniulida in their total evidence analysis, but only morphological characters could be scored for this enigmatic order. The group was recovered as sister to the remaining Helminthomorpha in both analyses.

 The remaining three orders (Spirobolida, Spirostreptida, and Julida) comprise the Juliformia. The Juliformia is based on the following synapomorphies: sternites fused to pleurotergites with suture present at interface, collum enlarged covering part of the head, and spermatozoa with pseudoperforatorium. The spirostreptid superfamily Cambalidea is of contentious placement and has been suggested to belong to the Julida. Alternatively, some authors have suggested the Cambalidea represents a unique 17^th^ order. The spirostreptid superfamily Epinannolenidea may also represent a unique order. We included two “Spirostreptida” species in this analysis: *Archispirostreptus gigas* (Spirostreptidea) and *Cambala* sp (Cambalidea). As a result, we cannot comment on the monophyly of the Juliformia or the Spirostreptida, but these superfamilies appear to have diverged between 200 and 300 MYA (Figure 4), on the order of other arthropod orders [70].

 Our results are perhaps most intuitive in regards to the placement of traditionally problematic taxa but differ from the most recent, and most inclusive, analysis of millipede ordinal phylogeny [2]. The placement of the Stemmiulida and Polydesmida are obvious points of disagreement between our analysis and that published by Sierwald and Bond, which placed the Stemmiulida sister to the Colobognatha+Eugnatha and the Polydesmida sister to the Colobognatha. This polydesmidan placement was obtained by Brewer et al. [32] when using full mitochondrial genomes to investigate millipede ordinal phylogenetics (stemmiulidans were not included in their analysis). Both Sierwald and Bond [2] and Brewer et al. [32] suffer from issues inherent to the data used to reconstruct their phylogenies. The former used the limited dataset, three nuclear protein coding genes, of Regier et al. [13] and the morphological matrix of Sierwald et al. [28]. In regards to Brewer et al. [32], one of the conclusions of the study was that mitochondrial genomes do not have adequate signal at timescales associated with millipede ordinal divergence. The placements of the Polydesmida and Stemmiulida, recovered as members of the Eugnatha, lend credence to the often discussed 8^th^ gonopod clade (Polydesmida + Stemmiulida + Chordeumatida + Callipodida) [71], though not in the strictest sense. The BI tree would support the existence of the 8^th^ gonopod clade in that the ancestor of the Eugnatha developed gonopods where the eighth leg pair transferred the spermatophore, and the Juliformia has a derived condition where the ninth leg pair is involved in sperm transfer (Figure 3). Again, topology comparisons preferred our results to previous hypotheses that were fully resolved with regards to our included taxa. 

### Molecular divergence dating

 The millipede fossil record, recently reviewed by Shear and Edgecombe [72], is extensive. The abundance of ancient diplopod fossils can be attributed, in large part, to the thick, calcified cuticle present in many chilognathan groups. The number of millipede fossils exceeds the numbers for the other myriapod classes by a large margin. Unfortunately, many of the fossil species are not placed in extant orders (especially the Paleozoic species), thus phylogenetic relationships to extant groups are ambiguous. Also, the Myriapoda and all of its daughter classes have presumably long “ghost lineages” (i.e., a lack of fossils showing transitional and marine forms that must have existed). As mentioned above, the oldest currently known land animal is a millipede, *Pneumodesmus newmani* Wilson and Anderson, 2004, living ~428 MYA that represents the derived helminthomorphan clade [9]. This coupled with the ambiguous placement of many millipede fossils makes using the diplopod fossil record to calibrate molecular clocks difficult. If the oldest fossil represents one of the most derived clades, other fossils underestimate the age of the many higher groups *de facto*. As a result, we used only *Pneumodesmus newmani* and *Sigmatria dilata* as calibration points for our estimates.

 As expected our divergence time estimations have tighter confidence intervals (CIs) at nodes closer to the calibration point. Deeper nodes have larger CIs and point estimates that are relatively old compared to recent studies focusing on higher taxonomic groups [61]. Rehm et al. [61], recently used the data and phylogeny of Meusemann et al. [64] to date the divergence times of major arthropod groups. The authors report the Chilopoda and Diplopoda split to have occurred 504 MYA using methods similar to those employed here. Overall, the dates recovered within the Diplopoda in both of our analyses are congruent. The lognormal method provided tighter CIs and more reasonable results for the deeper nodes. Regardless of the method, the millipede exemplars in this study represent lineages that originated long ago. These ancient divergence times are corroborated by the contemporary biogeography of many groups of millipedes. Recently reviewed by Shelley and Golovatch [6], many millipede orders anecdotally show current distributions that likely correspond to Pangaean, Gondwanan, or Laurasian origins.

 The Cambrian explosion, the observation that most modern animal body forms and phyla originated in a relatively short time period ~550 MYA, has received recent scrutiny. Possible metazoan trace fossils have been found dating as far back as 565 MYA [73]. Additionally, Erwin et al. [70] estimated the divergence dates within the Metazoa and found that animal life may have began diversifying in the Cryogenian over 700 MYA. These studies indicate that the Cambrian “explosion” may have been slower and began earlier than previously thought. Although the confidence intervals are relatively large (especially in the UGAM estimations), the ages recovered in our analyses, especially using the UGAM method, show ages of major arthropod lineages could be pre-Cambrian. Estimates of deep divergence times, especially nearing the root of trees, often have wide confidence intervals, and we are therefore conservative in our conclusions, particularly regarding the UGAM results.

## Conclusions

 Although our analyses lack sufficient sampling to warrant major nomenclatural changes concerning millipede groups above the ordinal level, the relationships we recovered have very strong support (all PP >/= 0.99). Additionally, this study includes an unprecedented amount of nuclear data for millipedes and more than most other arthropod studies as well. Moving forward, we will sequence more taxa and continue to expand and improve upon these data. The HaMSTR approach coupled with phylogenetic dataset selection and optimization, as outlined herein, appears to be very effective at selecting orthologous sequences and choosing amino acid residue characters with good signal at the depths we are investigating. These results are confidence inspiring and represent a step toward unraveling the relationships between higher-level millipede taxa, an achievement that has thus far proven difficult. Using this dataset containing many unlinked, protein-coding nuclear genes, some traditional groupings (e.g., the Nematorpha) are not recovered, whereas at least one as of yet unrecognized group is identified. Character systems once used to delineate major clades above the ordinal level (e.g., spinnerets) are suspect in light of these data, as are the timing of divergence between major groups (i.e., they appear to be more ancient than previously thought). Millipede high-level relationships have proven to be difficult to confidently reconstruct. Our data offers a much larger characters set than all other past analyses and presents novel results with high support values but these advantages are obviously countered with a paucity of taxa. Our results inspire confidence in a phylogenomic approach as we move forward collecting data for many more taxa representing orders not included here and lineages within each order. 

 These data used here to reconstruct the relationships between millipede taxa will likely also prove to be useful in many other ways. The relative completeness of transcriptome recovery in *Brachycybe lecontii* (93.95%) and other taxa will allow us to address many questions regarding millipede molecular evolution. Characterizations of the proteins found in various taxa including tests for selection, alternative splicing, and gene duplications are some of the additional areas we will address. As we continue to amass genomic-scale data, we will be able to investigate questions central to millipede character evolution, both morphological and molecular. For example, millipede defense secretions have been suggested to contain phylogenetic signal [4] and may show very interesting patterns of gain and loss in a complex set of characteristics. Future genomics based studies will continue to address important questions concerning millipede life history, physiology, and behavior, and a more taxonomically diverse phylogeny will be integral to studying the evolution of one of the planet’s oldest and most diverse land animal lineages.
